# Flow-Dependent Modulation of Endothelial Ca^2+^ Dynamics by Small Conductance Ca^2+^-Activated K^+^ Channels in Mouse Carotid Arteries

**DOI:** 10.3390/biomedicines12122900

**Published:** 2024-12-20

**Authors:** Mark S. Taylor, Michael Francis, Chung-Sik Choi

**Affiliations:** Department of Physiology and Cell Biology, University of South Alabama College of Medicine, Mobile, AL 36688, USA; michaelfrancis@southalabama.edu (M.F.); cchoi@southalabama.edu (C.-S.C.)

**Keywords:** endothelium, calcium, Ca^2+^ activated K^+^ channels, shear stress

## Abstract

Background: Small conductance Ca^2+^ activated K^+^ channels (K_Ca_2.3) are important regulators of vascular function. They provide Ca^2+^-dependent hyperpolarization of the endothelial membrane potential, promoting agonist-induced vasodilation. Another important mechanism of influence may occur through positive feedback regulation of endothelial Ca^2+^ signals, likely via amplification of influx through membrane cation channels. K_Ca_2.3 channels have recently been implicated in flow-mediated dilation of the arterial vasculature and may contribute to the crucial homeostatic role of shear stress in preventing vascular wall remodeling and progressive vascular disease (i.e., atherosclerosis). The impact of K_Ca_2.3 channels on endothelial Ca^2+^ signaling under physiologically relevant shear stress conditions remains unknown. Methods: In the current study, we employ mice expressing an endothelium-specific Ca^2+^ fluorophore (cdh5-GCaMP8) to characterize the K_Ca_2.3 channel influence on the dynamic Ca^2+^ signaling profile along the arterial endothelium in the presence and absence of shear-stress. Results: Our data indicate K_Ca_2.3 channels have a minimal influence on basal Ca^2+^ signaling in the carotid artery endothelium in the absence of flow, but they contribute substantially to amplification of Ca^2+^ dynamics in the presence of flow and their influence can be augmented through exogenous positive modulation. Conclusions: The findings suggest a pivotal role for K_Ca_2.3 channels in adjusting the profile of homeostatic dynamic Ca^2+^ signals along the arterial intima under flow.

## 1. Introduction

Localized dynamic Ca^2+^ signals provide a basis for functional signaling along the vascular intima [1]. We now know that spontaneous basal Ca^2+^ activity is an inherent property of the arterial endothelium, and these signals are expanded by various stimuli (e.g., Gq-coupled receptor agonists and cation channel stimulation) to elicit targeted and graded responses [2,3,4]. Expansion of specific dynamic Ca^2+^ signal parameters (i.e., event frequency, amplitude, duration, and spatial spread) underpins graded endothelium-dependent vasodilation in the resistance vasculature, impacting blood pressure and flow. Indeed, maintenance of cardiovascular homeostasis involves the titration of local Ca^2+^ signaling profiles by various local factors, circulating mediators, and physical forces along the vascular intima, including shear stress [5,6,7,8]. In addition to flow-mediated dilation of arteries, which promotes increased blood flow [9], fluid shear stress is crucial in conduit arteries for the preservation of vascular structure, and sustained low flow and oscillatory flow patterns promote progressive vascular remodeling and atherosclerosis [10,11,12,13,14]. In fact, disruption of flow alone (by approximately 90% for two weeks) in a carotid artery ligation mouse model causes progressive endothelial dysfunction and vascular remodeling, including occlusive neointimal formation within the lumen [15]. This pathological transition is associated with a distinct restriction of the dynamic Ca^2+^ signaling profile along the endothelial network, a pattern very similar to that observed downstream of occlusive lesions in artery branches from atherosclerotic patients [16].

Small and intermediate conductance Ca^2+^ activated potassium channels (K_Ca_) play a pivotal role in various tissues by controlling membrane potential [17,18,19]. In the vasculature, K_Ca_2.3 and K_Ca_3.1 isoforms are particularly important, responding to transient and local changes in endothelial Ca^2+^ flux as well as agonist-stimulated elevations in Ca^2+^ concentration to promote membrane potential hyperpolarization and vasorelaxation [2,20,21,22,23]. Vasorelaxation can occur through direct communication with vascular smooth muscle via myoendothelial gap junctions or through the release of mediators such as nitric oxide [24,25,26,27]. Within endothelial plasma membranes, K_Ca_2.3 channels cluster closely with cation channels, including vanilloid transient receptor type IV channels (TRPV4) [28,29]. This allows K_Ca_2.3 channels to not only hyperpolarize the membrane potential in response to Ca^2+^ influx (Ca^2+^-dependent K^+^ efflux) but also to increase the driving force for additional Ca^2+^ entry [30]. We previously showed that differential expression of K_Ca_2.3 channels can influence endothelial Ca^2+^ signals, particularly agonist-stimulated dynamic Ca^2+^ transients [31]. The implication is that this positive feedback augmentation of Ca^2+^ signals may act to further amplify endothelial responses. Recent findings suggest endothelial K_Ca_2.3 channels are important contributors to shear stress responses, including flow-mediated vasodilation of both resistance and conduit arteries, and may play a pivotal role in the preservation of vascular homeostasis [21,32]. This could have important implications not only for acute vasoregulation but also for chronic low-flow disease states such as atherosclerosis. Still, the specific impact of these channels on the endothelial Ca^2+^ signaling profile remains unclear.

Characterizing and understanding the complex Ca^2+^ dynamics driving diverse physiological endothelial functions will be crucial for resolving homeostatic endothelial signaling and defining early pathological transitions that progress to endothelial dysfunction and cardiovascular disease. We have recently implemented an extended custom analysis algorithm that allows for rigorous detection and quantification of the myriad signals occurring within intact tissues, including the endothelium [33]. In addition, incorporation of biosensor mice (cdh5-GCaMP8) that express a Ca^2+^-sensitive fluorophore exclusively within the endothelium has proven to be particularly valuable for discriminating endothelial signals in intact artery segments [34]. Here, we employ cdh5-GCaMP8 mice and our custom S8 analysis algorithm to assess the impact of K_Ca_2.3 channels on the carotid artery endothelial Ca^2+^ signaling profile under flow and no-flow conditions.

## 2. Materials and Methods

*Animals and tissue preparation.* Cdh5-GCaMP8 mice were bred and housed in the University of South Alabama College of Medicine vivarium. Mice (equal number male and female, 3–6 months, 25–35 g) were euthanized with pentobarbital sodium (50 mg/kg), and carotid arteries were removed and placed in (~4 °C) HEPES-buffered saline solution (HBSS) containing in mM, 134 NaCl, 6 KCl, 1 MgCl_2_, CaCl_2_, 10 HEPES, and 10 glucose; pH 7.45). All animal procedures were approved by the University of South Alabama Institutional Animal Care and Use Committee and carried out in accordance with the NIH Guide for the Care and Use of Laboratory Animals.

*Confocal imaging.* Artery segments were carefully cut open longitudinally and pinned down (with the endothelium facing up) using tungsten micropins in a 2 mm × 0.4 mm × 15 mm channel of a small silicone (sylgard) insert. Pinned arteries were stretched to 1.5 times their resting width, approximating vessel circumference at 80 mmHg as described previously [4]. The insert was placed in a 35 mL glass-bottom dish containing 300 µL HBSS connected to inflow/outflow lines and mounted on the stage of an Andor Revolution spinning disk confocal imaging system (inverted microscope, 20×). Ca^2+^-dependent fluorescence was measured at 7 frames/s at 25 °C using iQ software version 3.6.3 (1024 × 1024 pixels; 488 nm excitation, 510 nm emission). When indicated, flow was administered using a peristaltic pump connected to a reservoir of HBSS. Drugs or vehicles were added directly to the chamber (no flow) or via perfusate (flow). Recordings were acquired from intact intimal fields for at least 100 s periods and subjected to analysis offline.

*Ca^2+^ signal analysis.* 16-bit image sequence data were processed and analyzed using the S8 algorithm [33]. This python-based algorithm is designed to: (1) detect sites of dynamic Ca^2+^ change above statistical noise, (2) track dynamic regions of interest defined by the time course and footprint of individual events, and (3) quantify cumulative fluorescence activity (the dynamic signaling profile) by determining specific event parameters (frequency, amplitude, duration, and area). Cumulative area is the sum of maximal areas of all events occurring within a given recording and is used here as an index of total Ca^2+^ activity.

*Reagents and solutions.* Reagents and apamin were purchased from Sigma-Aldrich (St. Louis, MO, USA). N-cyclohexyl-2-(3,5-dimethylpyrazol-1-yl)-6-methylpyrimidin-4-amine (CyPPA) was purchased from Tocris (Minneapolis, MN, USA). Apamin was dissolved in HBSS, and CyPPA was dissolved in dimethyl sulfoxide (DMSO; final concentration 0.1%).

*Data Analysis.* Parameter distribution plots (amplitude, duration, and maximal area) and statistical analysis were performed using GraphPad Prism software (version 10.2.2). Data are presented as the mean ± standard error where appropriate. A paired *t*-test was used for comparisons before and after perturbation. N values represent the number of animals, and P-values less than 0.05 were considered significant.

## 3. Results

### 3.1. Endothelial Ca^2+^ Signaling Profile of cdh5-GCaMP8 Mouse Carotid Arteries Under No-Flow and Flow Conditions

**Figure 1:**
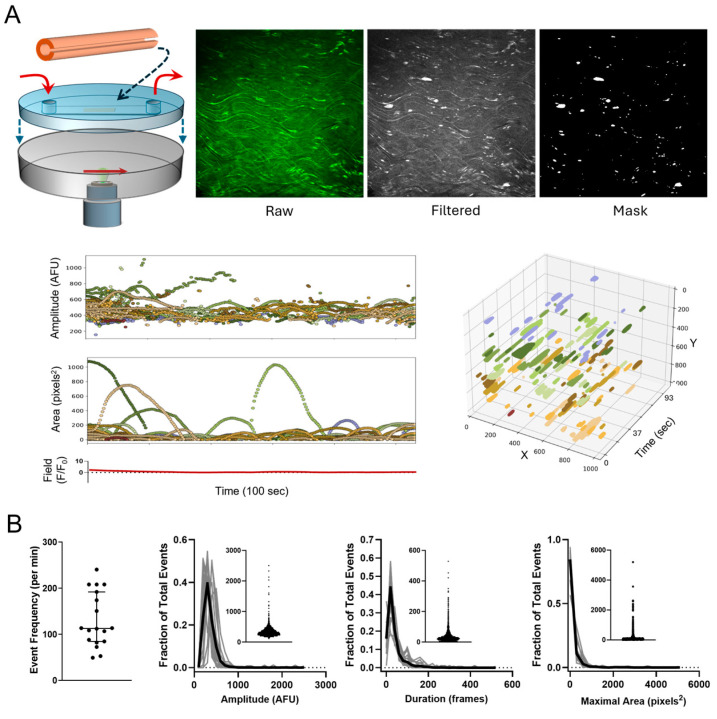

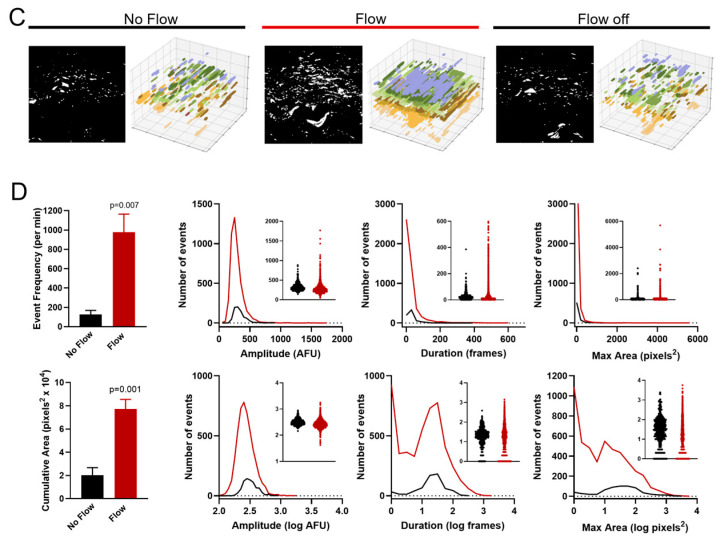
Characterization of dynamic Ca^2+^ signaling in cdh5-GCaMP8 mouse carotid artery endothelium. (**A**) Open carotid arteries were mounted in a flow chamber for fluorescence confocal imaging of the vascular endothelium under no-flow or flow conditions (red arrows). Images show isolation and tracking of basal dynamic Ca^2+^ signals from image sequences using S8 (under no-flow conditions). The three dimensional plot shows extrapolation of events in the field (x,y) over time (t). (**B**) Quantification of the basal Ca^2+^ signaling profile based on event frequency, amplitude, duration, and maximal area (18 arteries from 14 animals). (**C**) Characterization of Ca^2+^ dynamics in response to flow (10 dyn/cm^2^). (**D**) Summary of Ca^2+^ dynamics under flow (n = 4).

For these studies, we employed the cdh5-GCaMP8 mouse model that expresses a fluorescent endothelial Ca^2+^ sensor to assess dynamic endothelial Ca^2+^ transients along the intimal layer of carotid arteries (Figure 1A). We first characterized intrinsic endothelial Ca^2+^ signals under basal conditions (no flow). Applying our analysis platform S8 to raw image sequences, spatially and temporally discrete dynamic Ca^2+^ signals were isolated and tracked within continuous recordings and displayed as three-dimensional objects in a time-extrapolated plot (x, y, t). Assessment of these dynamic regions of interest allows for quantification of individual event parameters not discernible by tracking global mean fluorescence within the field (red line). Our data (Figure 1B) show that intrinsic dynamic Ca^2+^ events occurred at a median frequency of 113 per min within a 1.05 × 10^6^ pixel^2^ (4.4 × 10^5^ µm^2^) field of arterial intima. Histograms reveal right-skewed distributions of event parameters, including amplitude (median = 313 AFU), duration (median = 25 frames or 3.6 s), and maximal area (median = 35 pixels^2^ or 14.8 µm^2^). Next, we assessed the impact of flow on the endothelial Ca^2+^ signaling pattern. Figure 1C shows binary masks and corresponding time-extrapolated x–y plots in an endothelial field before flow, after introducing flow (shear stress 10 dyn/cm^2^), and after returning to no-flow conditions. Composite time-extrapolated plots of 100 s periods before, during, and after flow show rapid expansion of dynamic Ca^2+^ signals with the onset of flow that subside quickly after return to no-flow conditions. Of note, flow responses were measured 5 min after the onset of flow. As depicted in the summary plot (Figure 1D), increasing shear stress significantly increased the frequency of Ca^2+^ signals from 126 ± 24 to 977 ± 107 events/min, which included a marked expansion of new active sites along the intima. To further characterize the overall spatio-temporal footprint of the combined signals, we determined the cumulative maximal area of events in the field over the time course sampled, which increased almost four-fold from (2.0 ± 0.3) × 10^4^ to (7.7 ± 0.4) × 10^4^ pixels^2^ with the introduction of flow. Inspection of individual event parameter distributions shows that flow primarily expanded the major modes of event amplitude, duration, and maximal area that were already present under no-flow conditions. However, there was also a notable expansion of the range of event size and duration (evident in scatter plots) as well as a marked increase in the occurrence of very small transients as indicated by the left-most modes in log histograms of event duration and area. Overall, the data suggest that flow promotes expansion of Ca^2+^ signaling along the arterial intima by increasing the frequency of Ca^2+^ transients, including recruitment of new active sites, and establishes a profile of events along the intima distinct from the basal profile.

### 3.2. Effect of Small Conductance K_Ca_ Channel Inhibition on the Endothelial Ca^2+^ Signaling Profile Under No-Flow and Flow Conditions

**Figure 2:**
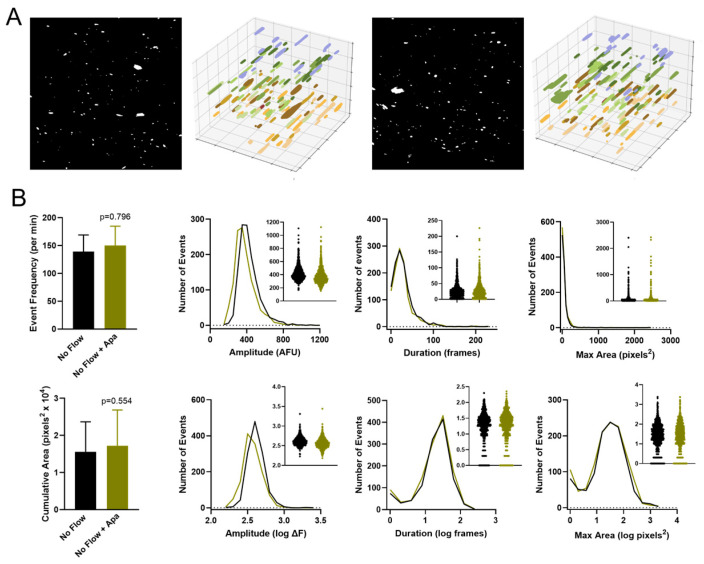
Effect of K_Ca_2.3 inhibition on endothelial Ca^2+^ dynamics under no-flow conditions. (**A**) Panels show binary masks and time-extrapolated plots of Ca^2+^ events before and after addition of apamin (0.5 µM) for 10 min. (**B**) Summary of Ca^2+^ dynamics before and after apamin (n = 5).

**Figure 3:**
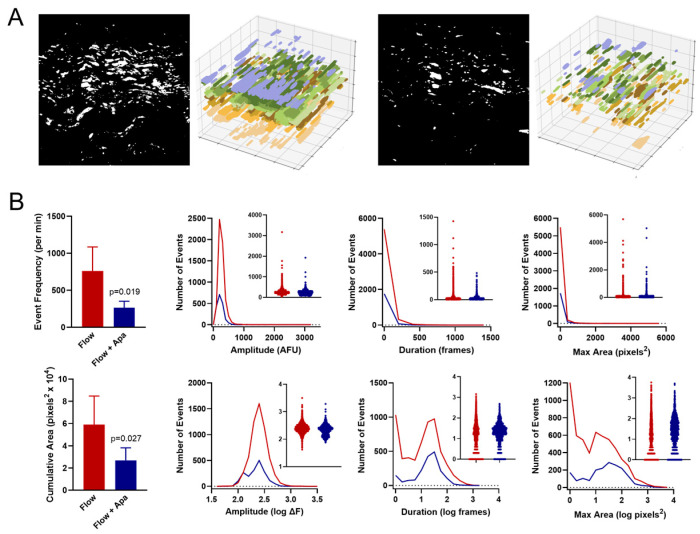
Effect of K_Ca_2.3 inhibition on endothelial Ca^2+^ dynamics under flow conditions. (**A**) Panels show binary masks and time-extrapolated plots of Ca^2+^ events at 10 dyn/cm^2^ shear stress before and after addition of apamin (0.5 µM) for 10 min. (**B**) Summary of Ca^2+^ dynamics before and after apamin (n = 5).

To determine the impact of small conductance K_Ca_ channels on endothelial Ca^2+^ dynamics under flow, we repeated measurements after the addition of the K_Ca_2.3 blocker apamin. Under no flow conditions, exposure to apamin (0.5 µM) had minimal effect on Ca^2+^ dynamics (Figure 2). Event frequency and cumulative maximal area were not significantly altered by apamin, and no substantial shift in event distribution was noted for event amplitude, duration, or maximal area. However, under flow conditions (10 dyn/cm^2^ for 5 min), the addition of apamin considerably impaired Ca^2+^ signaling (Figure 3), reducing event frequency from 763 ± 144 to 264 ± 39 events/min and reducing cumulative maximal area from (5.9 ± 1.1) × 10^4^ to (2.7 ± 0.5) × 10^4^ pixels^2^. This included a general reduction in individual Ca^2+^ event initiation sites within the field. Assessment of parameter distributions revealed a trend toward truncation of event size and duration and a notable suppression of the smallest Ca^2+^ transients as indicated by reduced peaks on the left side of duration and maximal area histograms.

### 3.3. Effect of Small Conductance K_Ca_ Channel Stimulation on the Endothelial Ca^2+^ Signaling Profile

**Figure 4:**
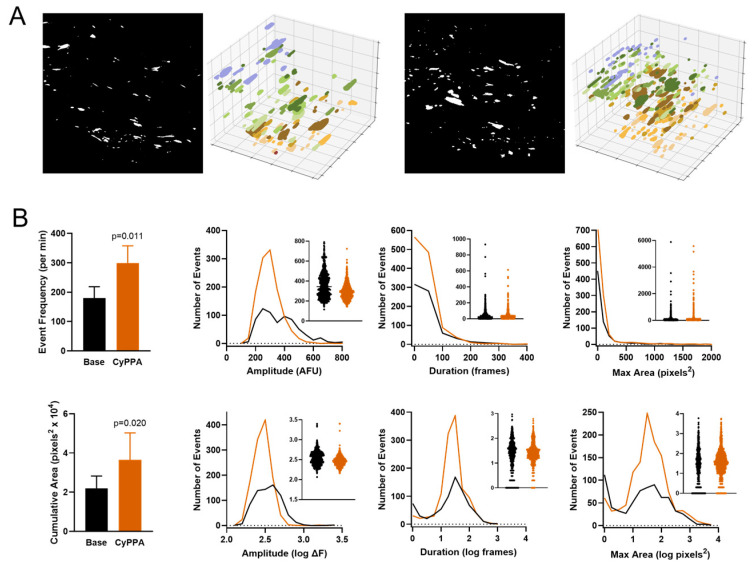

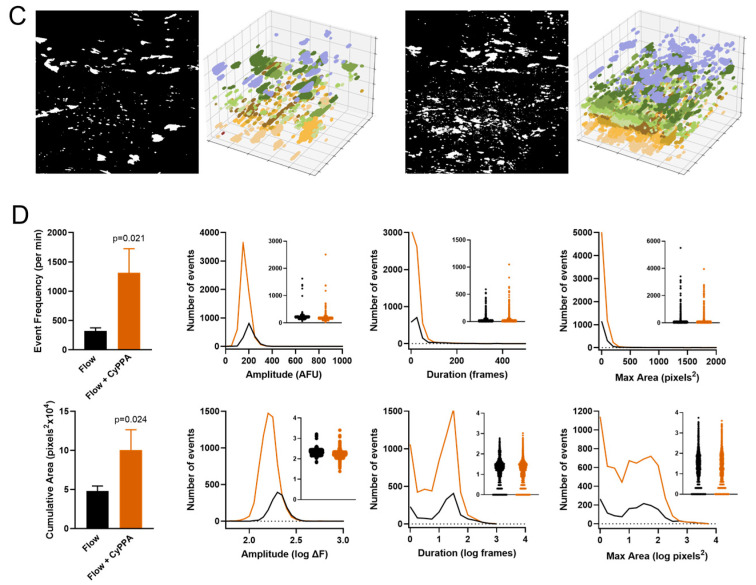
Effect of K_Ca_2.3 stimulation on endothelial Ca^2+^ dynamics under no-flow and flow conditions. (**A**) Panels show binary masks and time-extrapolated plots of Ca^2+^ events before and after addition of CyPPA (10 µM) for 10 min. (**B**) Summary of Ca^2+^ dynamics before and after CyPPA (n = 5). (**C**) Panels show binary masks and time-extrapolated plots of Ca^2+^ events at 5 dyn/cm^2^ shear stress before and after addition of CyPPA (10 µM) for 10 min. (**D**) Summary of Ca2+ dynamics before and after CyPPA (n = 4).

The cyclohexyl amine CyPPA acts as a positive modulator of K_Ca_2.3 channels by sensitizing their Ca^2+^-dependent activation [35]. Under no-flow conditions, the addition of CyPPA (10 µM) caused an increase in Ca^2+^ event frequency from 99 ± 14 to 152 ± 17 events/min (approximately 1.5-fold) and increased the cumulative maximal area of events from (1.7 ± 0.2) × 10^4^ to (2.6 ± 0.06) × 10^4^ pixels^2^ (Figure 4A,B). This augmented signaling included new initiation sites along the intima. Inspection of event parameter distributions revealed expansion of only the primary basal modes for amplitude, duration, and maximal area, indicating that direct stimulation of K_Ca_2.3 channels in the absence of flow mainly increases the occurrence of the most common (moderate-sized) endothelial Ca^2+^ events observed under basal conditions. Vehicle alone (0.1% DMSO) had no significant effect. Notably, CyPPA did not increase the occurrence of small Ca^2+^ transients or large Ca^2+^ waves. To determine whether the response to direct K_Ca_2.3 channel stimulation might be influenced by shear stress, we reassessed the effects of CyPPA under flow conditions (Figure 4C,D). For these experiments, we established constant flow at 5 dyn/cm^2^ for 5 min (half of the previous flow to ensure room for any event expansion) and then added CyPPA (10 µM) to the circulating perfusate. In the presence of flow, CyPPA substantially increased Ca^2+^ event frequency~4-fold from 320 ± 31 to 1313 ± 238 events/min and increased cumulative maximal area from (4.8 ± 0.4) × 10^4^ to (10.1 ± 1.5) × 10^4^ pixels^2^. This expansion included recruitment of new initiation sites. Overall, there was an increased occurrence of moderate-sized events as well as very small transients, as indicated by the expansion of the left-most modes of event duration and area distributions.

## 4. Discussion

Small conductance Ca^2+^ activated K^+^ channels (specifically the K_Ca_2.3 subtype) are pivotal regulators of cardiovascular function, eliciting Ca^2+^-dependent hyperpolarization of the vascular endothelium. These channels are known to be important contributors to agonist-induced vasodilation and have recently been implicated in shear stress-dependent responses [32], suggesting they may play an important role in sustaining cardiovascular homeostasis under physiological conditions. Our previous studies suggested that differential expression of K_Ca_2.3 channels can alter endothelial Ca^2+^ handling, and these channels may act as important amplifiers of Ca^2+^ signals controlling vascular function and structure. Here, we used cdh5-GCaMP mice expressing an endothelium-specific Ca^2+^ fluorophore and our analysis package S8 to characterize the influence of K_Ca_2.3 channels on the Ca^2+^ signaling profile of the carotid artery endothelium in the presence and absence of shear-stress. Our findings indicate that while K_Ca_2.3 channels appear to have little influence on the inherent basal Ca^2+^ signaling pattern under no-flow conditions, they play an important role in amplifying Ca^2+^ dynamics in the presence of sustained flow. This implies that K_Ca_2.3 channels may be integral regulators of homeostatic endothelial signaling and contribute to impaired signaling under low-flow conditions.

Key aspects of the current study include the use of the cdh5-GCaMP8 mouse for definitive isolation of endothelial Ca^2+^ dependent fluorescence and application of our analysis algorithm for automated detection and quantification of Ca^2+^ signaling profiles. In particular, the targeted endothelial expression of the cdh5-GCaMP8 biosensor reduces background fluorescence and avoids artifact signals from organelle compartmentalization and spill-over smooth muscle cell loading often seen with exogenous indicators. The S8 analysis algorithm allows for unbiased dynamic region of interest (ROI) tracking of fluorescence signals for multi-parameter characterization in space and time. We have found that application of S8 in cdh5-GCaMP8 tissues enhances signal-to-noise discrimination and quantitative tracking of dynamic Ca^2+^ signaling [33]. Also, in the current study, we assessed the same cellular fields before and after perturbation (such as flow), allowing us to determine both global signaling pattern changes and discrete cell/site recruitment changes within continuous endothelial fields. We chose to focus on carotid arteries for the current experiments since the findings are relevant to ongoing and future studies employing low-flow and atherosclerotic animal models such as the partial carotid artery ligation model [11,15].

Spontaneous dynamic endothelial Ca^2+^ signaling is an intrinsic feature of many vascular beds and species, including humans [1,16]. Indeed, inherent dynamic Ca^2+^ transients occurring along the arterial intima are constantly tuned by endothelial stimuli, directing the degree and specificity of vascular responses. Importantly, the recruitment and expansion of these dynamic Ca^2+^ transients, rather than global homogeneous rises in Ca^2+^ along the intima *per se* correspond to functional responses such as vasodilation [4,36]. In the current study, we found that the cdh5-GCaMP8 mouse carotid artery endothelium exhibited a basal Ca^2+^ signature that was consistent with that observed previously in wild-type mice, displaying similar event frequency and distinctive right-skewed parameter distributions [15]. Various mechanisms contributing to the detection and translation of shear stress along the vascular intima [8] act through regulation of endothelial cation/Ca^2+^ channels [37,38,39,40,41]. However, there is currently no consensus on a primary shear stress sensor or single mechanism. In fact, while some Ca^2+^ conducting channels may act as direct mechanosensors (including piezo 1 and TRP channel isoforms), others may be recruited through subsequent intracellular cascades and autocrine/paracrine signaling (e.g., ATP release and binding to P2RX4 receptors). Although the integration of this signaling is not yet clearly understood, the cellular effects are prolific and vital, ranging from immediate responses like vasodilation to extended effects on gene expression and phenotypic regulation. Notably, the current study was not designed to address specific conduits of shear-induced Ca^2+^ mobilization but rather to initially characterize the intrinsic Ca^2+^ patterning along the arterial intima with and without flow and determine whether K_Ca_2.3 channels contribute to modifying this Ca^2+^ profile. Here, we found that shear stress primarily expanded the existing Ca^2+^ profile by increasing the frequency of dynamic Ca^2+^ events along the intima. This included the recruitment of new cells and origination sites that were not active in the absence of flow, as well as the expansion of basally occurring signals. Notably, flow increased a wide range of both small and large events, which included a robust proliferation of localized transients of short duration as well as a population of long-lasting multicellular waves. These findings suggest that flow promotes a characteristic profile of Ca^2+^ signals along the arterial intima and that acute loss of shear stress restricts both the frequency and spatio-temporal profile of events.

A specific focus of the current study was whether K_Ca_2.3 channels might provide positive feedback control of the endothelial Ca^2+^ signals and what aspects of the signaling might be affected. Our findings point to a pivotal contribution of K_Ca_2.3 channels to the flow-mediated expansion of the intrinsic basal Ca^2+^ signaling profile. Specifically, K_Ca_2.3 channels appear to contribute to both a higher event frequency and an expanded range of Ca^2+^ event size and duration under flow conditions. It should be noted that direct stimulation of K_Ca_2.3 channels alone (via CyPPA) without flow also expanded the Ca^2+^ signaling profile, although it did not explicitly mimic the effect of flow; resulting in a <2-fold expansion of event frequency and cumulative area compared to a 4–6-fold increase elicited by flow. Most notably, direct K_Ca_2.3 stimulation did not cause the robust increase in small transients observed under flow conditions, supporting the idea that K_Ca_2.3 activation is not a primary mechanism of flow-mediated Ca^2+^ mobilization but rather acts as an amplifier. Consistent with this concept, when we re-assessed direct K_Ca_2.3 stimulation under conditions of flow, we observed very robust expansion of the flow-mediated Ca^2+^ dynamics that exceeded the relative impacts of K_Ca_2.3 stimulation without flow. Notably, under these conditions, K_Ca_2.3 stimulation increased both small and moderate sized events, suggesting these channels effectively amplify the distinctive shear stress Ca^2+^ profile. Overall, our data indicate that the influence of K_Ca_2.3 channels on the basal Ca^2+^ profile is essentially undetectable unless facilitated by Ca^2+^ sensitization, i.e., via CyPPA. However, under flow conditions, the shear stress-induced Ca^2+^ signals alone are capable of amplifying the K_Ca_2.3 influence, and this effect can be further expanded by exogenous Ca^2+^ sensitization of the channels.

The mechanism through which K_Ca_2.3 channels expand flow-mediated Ca^2+^ signals is unclear but may involve close association with membrane cation channels such as TRPV4 that are known to be activated in the presence of shear stress [42,43]. In this scenario, influx through TRPV4 channels could solicit Ca^2+^-dependent K^+^ efflux through proximal K_Ca_2.3 channels, enhancing the driving force for additional TRPV4 Ca^2+^ entry. These expanded Ca^2+^ signals at the membrane could also trigger Ca^2+^-induced Ca^2+^ release from inositol trisphosphate receptors (IP_3_Rs) on the endoplasmic reticulum, producing broader Ca^2+^ waves (perhaps accounting for the largest events with respect to event duration and area observed here). Indeed, broad cellular waves are triggered by stimulation of transient receptor potential ankyrin 1 (TRPA1) cation channels in rat cerebral artery endothelium [36] or TRPV4 channels in mouse mesenteric artery endothelium as long as internal stores are not depleted [3]. Release of Gq-protein-linked paracrine mediators like acetylcholine can also contribute to shear stress responses through IP_3_ production and sensitization of additional IP_3_R Ca^2+^ release [44]. It was recently reported that shear stress causes increased trafficking of K_Ca_2.3 channels to the plasma membrane of mesenteric artery endothelial cells [32]. This insertion of endosomes containing K_Ca_2.3 channels into the plasma membrane was shown to be dependent on TRPV4-mediated Ca^2+^ influx and Ca^2+^-dependent activation of the Rab GTPase Rab11A. Rab GTPases regulate anterograde and retrograde membrane trafficking in cells [45]. Overall, this resulted in increased K_Ca_2.3 channels clustered closely with TRPV4 channels in the plasma membrane. Such a scenario implies another level of positive feedback control of flow-dependent signaling whereby shear stress itself potentiates the K_Ca_2.3 channel impact on membrane potential and Ca^2+^ influx. Decreased K_Ca_2.3 channel activity has been implicated in endothelial dysfunction associated with hypertension and diabetes [46] and may play a key role in response to flow disruption and the development of atherosclerosis. We previously reported distinct truncation of the endothelial Ca^2+^ signaling pattern of conduit arteries under chronic low flow conditions that was associated with progressive endothelial dysfunction and vascular wall remodeling, including medial thickening and neointima formation [15]. This spatially and temporally restricted Ca^2+^ signaling profile mimics that observed in tibial artery branches downstream of occlusive atherosclerotic lesions in patients with peripheral artery disease [16]. Future work should identify key flow-linked elements coupled with K_Ca_2.3 channels and should address whether changes in K_Ca_2.3 expression and/or distribution along the arterial intima under conditions of compromised flow represent an early and pivotal pathological switch point contributing to the development of progressive vascular disease.

In the current study, we employed the cdh5-GCaMP8 mouse model and our analysis algorithm S8 to determine whether K_Ca_2.3 channels play a role in shaping the dynamic Ca^2+^ signaling profile of the carotid artery endothelium. We found that K_Ca_2.3 channels promote amplification of inherent endothelial Ca^2+^ dynamics under conditions of applied shear stress and may serve as important regulators of homeostatic Ca^2+^ signaling under physiologic flow.

## Data Availability

All data that support the findings from this study are available from the corresponding author upon reasonable request.
